# The diverse roles of FRO family metalloreductases in iron and copper homeostasis

**DOI:** 10.3389/fpls.2014.00100

**Published:** 2014-03-21

**Authors:** Anshika Jain, Grandon T. Wilson, Erin L. Connolly

**Affiliations:** Department of Biological Sciences, University of South CarolinaColumbia, SC, USA

**Keywords:** ferric reductase oxidase, metalloreductase, iron, copper, plant

## Abstract

Iron and copper are essential for plants and are important for the function of a number of protein complexes involved in photosynthesis and respiration. As the molecular mechanisms that control uptake, trafficking and storage of these nutrients emerge, the importance of metalloreductase-catalyzed reactions in iron and copper metabolism has become clear. This review focuses on the ferric reductase oxidase (FRO) family of metalloreductases in plants and highlights new insights into the roles of FRO family members in metal homeostasis. *Arabidopsis* FRO2 was first identified as the ferric chelate reductase that reduces ferric iron-chelates at the root surface-rhizosphere interface. The resulting ferrous iron is subsequently transported across the plasma membrane of root epidermal cells by the ferrous iron transporter, IRT1. Recent work has shown that two other members of the FRO family (FRO4 and FRO5) function redundantly to reduce copper to facilitate its uptake from the soil. In addition, FROs appear to play important roles in subcellular compartmentalization of iron as FRO7 is known to contribute to delivery of iron to chloroplasts while mitochondrial family members FRO3 and FRO8 are hypothesized to influence mitochondrial metal ion homeostasis. Finally, recent studies have underscored the importance of plasma membrane-localized ferric reductase activity in leaves for photosynthetic efficiency. Taken together, these studies highlight a number of diverse roles for FROs in both iron and copper metabolism in plants.

## INTRODUCTION

Iron (Fe) is essential for plants and is required for the function of a large number of enzymes involved in photosynthesis, respiration and a number of other processes. Iron’s utility in myriad biochemical processes stems from its ability to readily accept and donate electrons. It is most often associated with protein complexes either as a component of heme or Fe-S clusters. The ability of Fe to participate in electron transfer reactions is nevertheless problematic as well, since Fe^3^^+^ and Fe^2^^+^ are able to participate in the generation of the highly reactive hydroxyl radical (Halliwell and Gutteridge, 1992). As a result, it is critical that cells carefully control cellular Fe metabolism.

Iron limits plant growth in many soil types despite the fact that it is usually quite abundant. This is due to the fact that ferric iron is very poorly soluble in aerobic soils at neutral to basic pH. In the presence of oxygen, iron precipitates into insoluble Fe(III)-oxyhydroxide complexes. Thus, the molecular mechanisms utilized by plants for iron acquisition often include a first step that solubilizes ferric iron followed by a second step in which iron is transported from the soil and into root cells. Plants have evolved two types of strategies to combat iron deficiency. Strategy I is a reduction-based method used by all dicots and non-grass monocots while strategy II is used by grass species and involves chelation of ferric iron followed by uptake (Guerinot and Yi, 1994).

In response to iron deficiency, strategy I plants engage in a three stage process to acquire iron. First, the surrounding rhizosphere is acidified via proton extrusion by a root plasma membrane-localized proton ATPase, AHA2 (*Arabidopsis* H^+^ATPase 2; Santi and Schmidt, 2009). This serves to increase solubility of ferric iron complexes. Ferric iron chelates are then reduced to ferrous iron by FRO2 (ferric reductase oxidase 2) and Fe^2^^+^ ions are subsequently taken up into root cells by the divalent metal transporter, IRT1 (iron regulated transporter 1; Eide et al., 1996; Yi and Guerinot, 1996; Robinson et al., 1999; Vert et al., 2002). In contrast, strategy II plants secrete phytosiderophores (PSs), such as mugineic acid, which bind to ferric iron with high affinity (Walker and Connolly, 2008). The resulting Fe(III)-PS complexes are transported across the root plasma membrane via the yellow stripe1 (YS1) iron transporter (Curie et al., 2001).

In this review, we focus on the roles of the FRO family of metalloreductases in reduction of iron and copper in plants. To this end, we briefly review what is known about reduction of iron at the root surface and highlight new work that has demonstrated a role for FRO family members in reduction of copper for uptake by plants. In addition, we focus on the emerging roles of FROs in trafficking of iron to subcellular compartments.

## THE FRO FAMILY OF METALLOREDUCTASES

The reduction of ferric iron to ferrous iron at the root surface is a process that has been well documented and characterized across several plant species including *Arabidopsis *(Yi and Guerinot, 1996), pea (Waters et al., 2002), and tomato (Li et al., 2004), as well as the green alga *Chlamydomonas* *reinhardtii *(Eckhardt and Buckhout, 1998). The first plant metalloreductase gene was cloned from *Arabidopsis* (Robinson et al., 1999). FRO2 was identified based on its sequence similarity to the yeast ferric reductase, FRE1, as well as to a subunit of the human NADPH oxidase, gp91phox, which is involved in the production of reactive oxygen species to protect against invading pathogens (Robinson et al., 1999; Vignais, 2002). FRO2 was shown to complement the phenotype of an *Arabidopsis* ferric**reductase defective-1 mutant (*frd1*), thus proving that *FRO2* encodes the root surface ferric chelate reductase. As expected for an enzyme involved in iron acquisition from the soil, *FRO2* is expressed in the root epidermis and is strongly induced by iron limitation (Connolly et al., 2003). Constitutive high-level expression of *FRO2* in soybean confers enhanced tolerance to iron deficiency-induced chlorosis (Vasconcelos et al., 2006).

FRO2 belongs to a superfamily of flavocytochromes and is involved in transfer of electrons from the cytosol across the plasma membrane to reduce extracellular ferric iron to ferrous iron. Studies of the topology of FRO2 show that the protein contains eight transmembrane (TM) helices, four of which comprise the highly conserved core of the protein (Schagerlof et al., 2006). This core is conserved throughout the flavocytochrome b family. The large water-soluble domain of FRO2, which contains NADPH, flavin adenine dinucleotide (FAD), and oxidoreductase sequence motifs, is located in the cytosol. FRO2 also contains four highly conserved histidine residues that likely coordinate two intramembranous heme groups that are instrumental in the electron transfer process (Robinson et al., 1999; Schagerlof et al., 2006). Although FRO2 appears to be solely responsible for reduction of ferric iron chelates in the rhizosphere, the *Arabidopsis* genome encodes a total of eight FRO family members. The seven additional FRO proteins are believed to function as metalloreductases primarily involved in the reduction of iron and possibly copper; here, we highlight new insight into the roles of FRO family members in copper reduction and intracellular metal trafficking.

## PLASMA MEMBRANE-LOCALIZED ROOT COPPER REDUCTASES

Studies of the yeast FRE family have uncovered roles for these proteins in reduction of both iron and copper (Hassett and Kosman, 1995; Georgatsou et al., 1997; Martins et al., 1998). Consistent with their roles in the high-affinity iron and copper uptake systems, their expression is regulated by both iron and copper status. Like their FRE counterparts, *Arabidopsis* *FRO* genes are differentially regulated by deficiencies of iron and/or copper (Mukherjee et al., 2006). Studies of FRO2 have suggested that it may have a role in the reduction of Cu^2^^+^ to Cu^+^ at the root surface, in addition to its role in iron reduction (Yi and Guerinot, 1996; Robinson et al., 1999). *Arabidopsis* plants show an increase in root copper reductase activity under iron limitation and *frd1* mutants fail to induce this activity in response to iron limitation (Robinson et al., 1999). However, copper concentrations are not reduced in *frd1* mutants, suggesting that reduction of copper by FRO2 is not physiologically relevant; this result opens up the possibility that other FROs function to reduce copper at the root surface. It is possible that copper uptake may proceed without prior reduction of Cu^2^^+^ to Cu^+^, perhaps via a ZRT, IRT-like protein (ZIP)-type transporter. Interestingly, expression of *ZIP2* and *ZIP4* is upregulated under copper limitation (Wintz et al., 2003). However, stable isotope studies support a reduction-based pathway for copper uptake (Jouvin et al., 2012). Indeed, recent studies have shown that FRO4 and FRO5 act redundantly to reduce copper at the root surface (Bernal et al., 2012)

The SPL7 (SQUAMOSA promoter binding-like7) transcription factor functions as a master regulator of the copper deficiency response in *Arabidopsis* (Yamasaki et al., 2009). Recently, RNA-Seq revealed that *FRO4* and *FRO5* are strongly upregulated in roots under copper limitation. In addition, induction of *FRO4* and *FRO5* in roots under copper limitation depends on SPL7 (Bernal et al., 2012). *FRO4* and *FRO5 *lie in tandem on chromosome 5 and share high sequence similarity at the amino acid level (Mukherjee et al., 2006). SPL7 has been shown to bind to a CuRE (Cu responsive element) in promoters of copper regulated genes (Yamasaki et al., 2004; Yamasaki et al., 2009) similar to its homolog in *C. reinhardtii*, CCR1 (COPPER RESPONSIVE REGULATOR1; Quinn and Merchant, 1995; Kropat et al., 2005; Sommer et al., 2010). *FRO4* and *FRO5* each contain GTAC motifs in their upstream promoter regions, suggesting that they may be direct targets of SPL7 (Bernal et al., 2012). *fro4*, *fro5,* and *fro4fro5* double mutant lines display significant decreases in copper deficiency-inducible copper reductase activity. In addition, use of a fluorescent dye [coppersensor-1 (CS1)] that binds Cu^+^ showed that uptake of Cu^+^ in the *fro4* and *fro5* single mutants was markedly lower than in wild type plants and *fro4fro5* double mutant plants show hardly any detectable Cu^+^, demonstrating that FRO4 and FRO5 function redundantly as copper reductases in the high affinity copper uptake pathway (Bernal et al., 2012). In addition, although *spl7* plants lack expression of *FRO4* and *FRO5* and corresponding Cu-deficiency inducible root Cu reductase activity, *spl7* does display elevated *FRO2* transcript abundance and root ferric chelate reductase activity. These results clearly establish that FRO4 and FRO5 (rather than FRO2) are responsible for reduction of Cu at the root surface (Bernal et al., 2012). It remains unclear whether FRO4 and FRO5 are involved in Fe homeostasis, however, expression of *FRO5* is induced under iron deficiency (Wu et al., 2005; Mukherjee et al., 2006).

## PUTATIVE PLASMA MEMBRANE-LOCALIZED LEAF FERRIC REDUCTASE

Following uptake from the soil, iron must be loaded into the xylem, where it is found as a ferric-citrate complex (Rellan-Alvarez et al., 2010). How iron is transported into leaf cells remains unknown, but it is thought that Fe(III)-chelates may need to be reduced prior to transport into leaf cells. *FRO6* is expressed at high levels in leaves (Mukherjee et al., 2006), and overexpression of *FRO6* in tobacco showed that *FRO6* can facilitate the reduction of iron in leaves (Li et al., 2011). *FRO6* expression is not affected by iron status (Mukherjee et al., 2006). Instead, analysis of *FRO6-GUS* lines has shown that *FRO6* expression is controlled in a light-dependent manner. Indeed, the *FRO6 *promoter contains several light-responsive elements and etiolated *FRO6-GUS* seedlings exhibit no *FRO6* promoter activity (Feng et al., 2006). Together, these data suggest that FRO6 may function to reduce iron in leaves when light is available, perhaps to enable the assembly of new photosynthetic complexes.

## INTRACELLULAR METALLOREDUCTASES

Chloroplasts and mitochondria represent significant sinks for Fe. Indeed, the vast majority of Fe found within leaves is located within chloroplasts. Essential cofactors such as heme and Fe–S clusters are synthesized in chloroplasts and mitochondria. Despite this, the molecular mechanisms by which iron is trafficked to these two organelles are not well understood. Recent studies implicate FRO family members in iron delivery to chloroplasts and mitochondria. Intriguingly, although work in yeast has shown that metalloreductases are important in vacuolar metal homeostasis, to date there is no evidence to support an analogous role in plants.

## CHLOROPLASTIC FERRIC REDUCTASE

Although the precise mechanisms involved in chloroplast iron acquisition are still somewhat murky (Landsberg, 1984; Terry and Abadia, 1986; Bughio et al., 1997; Shikanai et al., 2003), it seems likely that chloroplasts take up both Fe(II) and Fe(III) via multiple pathways as observed in modern day cyanobacteria. Free living cyanobacteria have been shown to acquire iron through Fe^2^^+^ iron transporters from a pool of Fe(III)-dicitrate complexes (Katoh et al., 2001) and it is thus clear that some species of cyanobacteria are able to use a reduction-based mechanism for iron uptake (Kranzler et al., 2014). Plant chloroplasts, which are thought to have originated from ancient cyanobacteria, appear to utilize a similar strategy for iron uptake as studies of *Arabidopsis* FRO7 demonstrate that chloroplasts employ a reduction-based strategy for iron acquisition. FRO7 localizes to chloroplasts and loss of FRO7 function results in a significant reduction in chloroplast surface ferric reductase activity. In addition, *fro7* chloroplasts show a ~30% reduction in chloroplast Fe content. *fro7* grows poorly on medium lacking sucrose and shows reduced photosynthetic efficiency, consistent with the idea that FRO7 is critical for delivery of Fe for proper assembly of photosynthetic complexes. When sown on alkaline soil, *fro7 *seeds germinate but the resulting seedlings are severely chlorotic and the plants fail to set seed unless supplemented with excess iron (Jeong et al., 2008). Recent work in sugar beet further supports the existence of a reduction-based mechanism for iron uptake by chloroplasts, as well (Solti et al., 2012).

A presumptive Fe transporter, PIC1, has been identified that localizes to the chloroplast envelope (Duy et al., 2007). Whether FRO7 and PIC1 work together in chloroplast iron uptake currently remains unknown and it is not yet clear whether PIC1 transports ferric or ferrous iron. Other proteins that are presumed to function in chloroplast Fe transport are MAR1 (a homolog of ferroportin 1 and 2), which may transport an iron chelator (Conte et al., 2009), MFL1/2 [which resemble mitoferrins but function in chloroplasts; (Tarantino et al., 2011) and NAP14 (Shimoni-Shor et al., 2010)]. In addition, a chloroplast-and mitochondria-localized NEET-type protein was recently identified which may be involved in Fe–S cluster transfer to apoproteins (Nechushtai et al., 2012).

## PUTATIVE MITOCHONDRIAL FERRIC REDUCTASES

Studies in *Arabidopsis* have identified a putative iron-chaperone (Busi et al., 2006; Vazzola et al., 2007) and putative mitochondrial effluxer proteins involved in iron metabolism (Kushnir et al., 2001; Chen et al., 2007). In addition, a recent report described the identification of a mitochondrial Fe transporter in rice (MIT1) which is essential for plant growth (Bashir et al., 2011). Despite this, we are far from a comprehensive understanding of mitochondrial Fe homeostasis (Nouet et al., 2011; Vigani et al., 2013). Although two *Arabidopsis* metalloreductases (FRO3 and FRO8) have been predicted to localize to mitochondrial membranes, neither one has been functionally characterized. A mitochondrial proteomics study has placed FRO8 at the mitochondrial membrane (Heazlewood et al., 2004). The expression patterns of *FRO3* and *FRO8* are largely non-overlapping, suggesting that they do not function redundantly (Jain and Connolly, 2013). Little information is available for *FRO8* but its expression is concentrated in the vasculature of senescing leaves (Wu et al., 2005). *FRO3* is expressed most highly in the vasculature of young seedlings and its expression is strongly induced under iron deficiency; for this reason, *FRO3* has been widely used as an iron deficiency marker (Mukherjee et al., 2006; Tarantino et al., 2010). Interestingly, *FRO3* expression is negatively regulated by the basic helix loop helix (bHLH) transcription factor PYE (POPEYE); PYE appears to control a pericycle-specific Fe deficiency response in roots (Dinneny et al., 2008; Long et al., 2010). *FRO3* expression also is responsive to copper status (Mukherjee et al., 2006; Yamasaki et al., 2009). Despite this, the roles of FRO3 and FRO8 remain unclear. It is interesting to note that although the yeast metalloreductase FRE5 localizes to mitochondria (Sickmann et al., 2003), there are no reports to date that demonstrate a role for a metalloreductase in mitochondria in any organism.

## VACUOLAR IRON TRAFFICKING

Acidic compartments like vacuoles have a relatively oxidizing atmosphere as compared to the cytosol. In yeast, iron in vacuoles is largely present as ferric polyphosphate complexes (Raguzzi et al., 1988). The remobilization of iron from the yeast vacuolar compartment is mediated by the FRE6 ferric chelate reductase (Singh et al., 2007). FRE6 also plays a role in copper remobilization from vacuoles; reduced copper is subsequently exported to the cytosol via CTR2 (copper transporter 2; Rees and Thiele, 2007). Vacuolar iron transporters have been reported in plants; *Arabidopsis* vacuolar iron transporter (VIT1), transports iron into the organelle while NRAMP3 (natural resistance against microbial pathogens3) and NRAMP4 mediate the export of iron (Lanquar et al., 2005; Kim et al., 2006). However, no vacuolar metalloreductases have been reported in plants, to date.

## CONCLUSION

**FIGURE 1:**
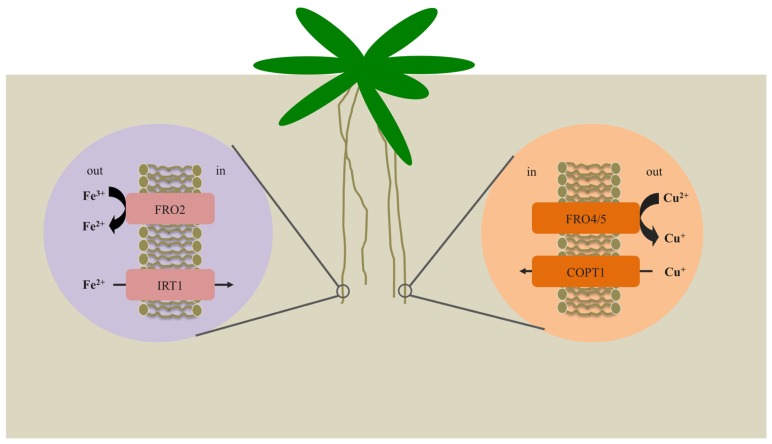
**Mechanisms for iron and copper uptake by *Arabidopsis* roots**. Under iron-deficient conditions, expression of *FRO2* and *IRT1* is enhanced. FRO2 serves to reduce solubilized Fe^3^^+^ to Fe^2^^+^, which is then transported across the root plasma membrane via IRT1. Under copper-deficient conditions, FRO4 and FRO5 are highly expressed in the roots and function to reduce Cu^2^^+^ to Cu^+^ prior to uptake by COPT1.

**FIGURE 2:**
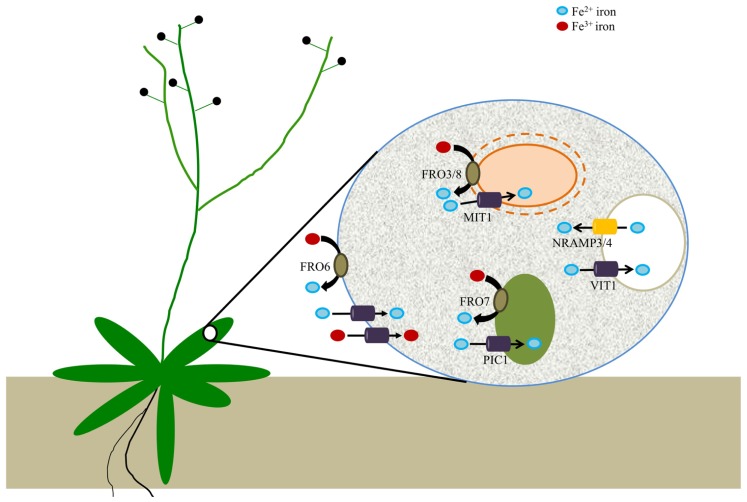
***Arabidopsis* ferric reductases and transporters that contribute to cellular iron homeostasis**. Evidence suggests that FRO6 functions to reduce Fe^3^^+^ to Fe^2^^+^ at the cell surface of leaf cells; Fe^2^^+^ is subsequently transported across the membrane via an unknown transporter(s), while other unknown transporters may be involved in the uptake of Fe^3^^+^. Iron is then trafficked to a set of intracellular organelles. Chloroplasts utilize a reduction-based mechanism for iron acquisition via FRO7, whereas FRO3 and FRO8 may serve an analogous function in mitochondria. PIC1 serves as a chloroplast iron transporter while rice MIT mediates iron uptake by mitochondria. Although there is not yet any evidence for vacuolar metalloreductases in plants, it is known that VIT1 is important for iron uptake by vacuoles while *NRAMP3/4* function in vacuolar Fe efflux.

Plants require iron and copper for vital processes such as photosynthesis, respiration, and nitrogen fixation. While it has been known for some time that ferric chelate reductases play a vital role in iron uptake from the soil by all plant species except for grasses, other roles for FROs in metal homeostasis have only recently emerged. Indeed, new studies have shown that FROs are important for copper acquisition from the soil (**Figure 1**) and for intracellular distribution of Fe (**Figure 2**). Together, these studies have shed considerable light on the molecular mechanisms employed by plants to maintain Fe and Cu homeostasis. In addition, this new knowledge should facilitate novel strategies aimed at improving crop yields on nutrient-poor soils and biofortification of plant foods to help ameliorate nutrient deficiencies in humans. Future studies will likely focus on the precise roles of mitochondrial FROs in mitochondrial metal metabolism. Furthermore, our understanding of iron trafficking within cells is severely hampered by our limited understanding of the various subcellular iron pools. New tools that provide insight into the redox status and types of iron species found in each of the various cellular compartments will go a long way toward the development of a comprehensive understanding of iron metabolism in plants.

## Conflict of Interest Statement

The authors declare that the research was conducted in the absence of any commercial or financial relationships that could be construed as a potential conflict of interest.

## References

[B1] Bashir K. Ishimaru Y. Shimo H. Nagasaka S. Fujimoto M. Takanashi H. (2011). The rice mitochondrial iron transporter is essential for plant growth. *Nat. Commun.* 2 1–7 10.1038/ncomms1326 PMC3113228 21610725

[B2] Bernal M. Casero D. Singh V. Wilson G. T. Grande A. Yang H. (2012). Transcriptome sequencing identifies SPL7-regulated Cu acquisition genes FRO4/FRO5 and the Cu dependence of Fe homeostasis in *Arabidopsis*. *Plant Cell* 24 738–761 10.1105/tpc.111.090431 22374396 PMC3315244

[B3] Bughio N. Takahashi M. Yoshimura E. Nishizawa N. K. Mori S. (1997). Light-dependent iron transport into isolated barley chloroplasts. *Plant Cell Physiol.* 38 101–105 10.1093/oxfordjournals.pcp.a029079

[B4] Busi M. V. Maliandi M. V. Valdez H. Clemente M. Zabaleta E. J. Araya A. (2006). Deficiency of *Arabidopsis thaliana *frataxin alters activity of mitochondrial Fe-S proteins and induces oxidative stress. *Plant J.* 48 873–882 10.1111/j.1365-313X.2006.02923.x 17092311

[B5] Chen S. Sanchez-Fernandez R. Lyver E. R. Dancis A. Rea P. A. (2007). Functional characterization of AtATM1, AtATM2 and AtATM3, a subfamily of *Arabidopsis* half-molecule ABC transporters implicated in iron homeostasis. *J. Biol. Chem.* 282 21561–21571 10.1074/jbc.M702383200 17517886

[B6] Connolly E. L. Campbell N. Grotz N. Prichard C. L. Guerinot M. L. (2003). Overexpression of the FRO2 iron reductase confers tolerance to growth on low iron and uncovers post-transcriptional control. *Plant Physiol.* 133 1102–1110 10.1104/pp.103.025122 14526117 PMC281606

[B7] Conte S. Stevenson D. Furner I. Lloyd A. (2009). Multiple antibiotic resistance in *Arabidopsis* is conferred by mutations in a chloroplast-localized transport protein. *Plant Physiol.* 151 559–573 10.1104/pp.109.143487 19675150 PMC2754617

[B8] Curie C. Panaviene Z. Loulergue C. Dellaporta S. L. Briat J. F. Walker E. L. (2001). Maize yellow stripe1 encodes a membrane protein directly involved in Fe(III) uptake. *Nature* 409 346–349 10.1038/35053080 11201743

[B9] Dinneny J. R. Long T. A. Wang J. Y. Jung J. W. Mace D. Pointer S. (2008). Cell identity mediates the response of *Arabidopsis* roots to abiotic stress. *Science* 320 942–945 10.1126/science.1153795 18436742

[B10] Duy D. Wanner G. Meda A. R. Von Wiren N. Soll J. Philippar K. (2007). PIC1, an ancient permease in *Arabidopsis* chloroplasts, mediates iron transport. *Plant Cell* 19 986–1006 10.1105/tpc.106.047407 17337631 PMC1867359

[B11] Eckhardt U. Buckhout T. J. (1998). Iron assimilation in *Chlamydomonas reinhardtii* involves ferric reduction and is similar to strategy I higher plants. *J. Exp. Bot.* 9 1219–1226

[B12] Eide D. Broderius M. Fett J. Guerinot M. L. (1996). A novel iron-regulated metal transporter from plants identified by functional expression in yeast. *Proc. Natl. Acad. Sci. U.S.A.* 93 5624–5628 10.1073/pnas.93.11.5624 8643627 PMC39298

[B13] Feng H. An F. Zhang S. Ji Z. Ling H. Q. Zuo J. (2006). Light-regulated, tissue-specific, and cell differentiation-specific expression of the *Arabidopsis* Fe(III)-chelate reductase gene AtFRO6. *Plant Physiol.* 140 1345–1354 10.1104/pp.105.074138I 16489134 PMC1435798

[B14] Georgatsou E. Mavrogiannis L. A. Fragiadakis G. S. Alexandraki D. (1997). The yeast Fre1p/Fre2p cupric reductases facilitate copper uptake and are regulated by the copper-modulated Mac1p activator. *J. Biol. Chem.* 272 13786–13792 10.1074/jbc.272.21.13786 9153234

[B15] Guerinot M. L. Yi Y. (1994). Iron: nutritious, noxious, and not readily available. *Plant Physiol.* 104 815–820 10.1104/pp.104.3.815 12232127 PMC160677

[B16] Halliwell B Gutteridge J. M. C. (1992). Biologically relevant metal ion-dependent hydroxyl radical generation. *FEBS Lett.* 307 108–112 10.1016/0014-5793(92)80911-Y 1322323

[B17] Hassett R. Kosman D. J. (1995). Evidence for Cu(II) reduction as a component of copper uptake by *Saccharomyces cerevisiae*. *J. Biol. Chem.* 270 128–134 10.1074/jbc.270.1.128 7814363

[B18] Heazlewood J. L. Tonti-Filippini J. S. Gout A. M. Day D. A. Whelan J. Millar A. H. (2004). Experimental analysis of the *Arabidopsis* mitochondrial proteome highlights signaling and regulatory components, provides assessment of targeting prediction programs, and indicates plant-specific mitochondrial proteins. *Plant Cell* 16 241–256 10.1105/tpc.016055 14671022 PMC301408

[B19] Jain A. Connolly E. L. (2013). Mitochondrial iron transport and homeostasis in plants. *Front. Plant Sci.* 4: 348 10.3389/fpls.2013.00348 PMC3764374 24046773

[B20] Jeong J. Cohu C. Kerkeb L. Pilon M. Connolly E. L. Guerinot M. L. (2008). Chloroplast Fe(III) chelate reductase activity is essential for seedling viability under iron limiting conditions. *Proc. Natl. Acad. Sci. U.S.A.* 105 10619–10624 10.1073/pnas.0708367105 18647837 PMC2492473

[B21] Jouvin D. Weiss D. J. Mason T. F. Bravin M. N. Louvat P. Zhao F. (2012). Stable isotopes of Cu and Zn in higher plants: evidence for Cu reduction at the root surface and two conceptual models for isotopic fractionation processes. *Environ. Sci. Technol.* 46 2652–2660 10.1021/es202587m 22296233

[B22] Katoh H. Hagino N. Grossman A. R. Ogawa T. (2001). Genes essential to iron transport in the cyanobacterium*Synechocystis* sp. *strain PCC *6803. *J. Bacteriol.* 183 2779–2784 10.1128/JB.183.9.2779-2784.2001 PMC99493 11292796

[B23] Kim S. A. Punshon T. Lanzirotti A. Li L. Alonso J. M. Ecker J. R. (2006). Localization of iron in *Arabidopsis* seed requires the vacuolar membrane transporter VIT1. *Science* 314 1295–1298 10.1126/science.1132563 17082420

[B24] Kranzler C. Lis H. Finkel O. M. Schmetterer G. Shaked Y. Keren N. (2014). Coordinated transporter activity shapes high-affinity iron acquisition in cyanobacteria. *ISME J.* 8 409–417 10.1038/ismej.2013.161 24088625 PMC3906821

[B25] Kropat J. Tottey S. Birkenbihl R. P. Depege N. Huijser P. Merchant S. (2005). A regulator of nutritional copper signaling in Chlamydomonas is an SBP domain protein that recognizes the GTAC core of copper response element. *Proc. Natl. Acad. Sci. U.S.A.* 102 18730–18735 10.1073/pnas.0507693102 16352720 PMC1311908

[B26] Kushnir S. Babiychuk E. Storozhenko S. Davey M. W. Papenbrock J. De Rucke R. (2001). A mutation of the mitochondrial ABC transporter Sta1 leads to dwarfism and chlorosis in the *Arabidopsis* mutant starik. *Plant Cell* 13 89–100 11158531 10.1105/tpc.13.1.89 PMC102216

[B27] Landsberg E. (1984). Regulation of iron-stress-response by whole-plant activity. *J. Plant Nutr.* 7 609–621 10.1080/01904168409363226

[B28] Lanquar V. Lelievre F. Bolte S. Hames C. Alcon C. Neumann D. (2005). Mobilization of vacuolar iron by AtNRAMP3 and AtNRAMP4 is essential for seed germination on low iron. *EMBO J.* 24 4041–4051 10.1038/sj.emboj.7600864 16270029 PMC1356305

[B29] Li L. Cheng X. Ling H. Q. (2004). Isolation and characterization of Fe(III)-chelate reductase gene LeFRO1 in tomato. *Plant Mol. Biol.* 54 125–136 10.1023/B:PLAN.0000028774.82782.16 15159639

[B30] Li L. Y. Cai Q. Y. Yu D. S. Guo C. H. (2011). Overexpression of AtFRO6 in transgenic tobacco enhances ferric chelate reductase activity in leaves and increases tolerance to iron-deficiency chlorosis. *Mol. Biol. Rep.* 38 3605–3613 10.1007/s11033-010-0472-9I 21104018

[B31] Long T. A. Tsukagoshi H. Busch W. Lahner B. Salt D. E. Benfey P. N. (2010). The bHLH transcription factor POPEYE regulates response to iron deficiency in *Arabidopsis* roots. *Plant Cell* 22 2219–2236 10.1105/tpc.110.074096 20675571 PMC2929094

[B32] Martins L. J. Jensen L. T. Simon J.R. Keller G. L. Winge D. R. (1998). Metalloregulation of FRE1 and FRE2 homologs in *Saccharomyces cerevisiae*. *J. Biol. Chem.* 273 23716–23721 10.1074/jbc.273.37.23716 9726978

[B33] Mukherjee I. Campbell N. H. Ash J. S. Connolly E. L. (2006). Expression profiling of the *Arabidopsis* ferric chelate reductase (*FRO*) gene family reveals differential regulation by iron and copper. *Planta* 223 1178–1190 10.1007/s00425-005-0165-0 16362328

[B34] Nechushtai R. Conlan A. R. Harir Y. Song L. Yogev O. Eisenberg-Domovich Y. (2012). Characterization of *Arabidopsis* NEET reveals an ancient role for NEET proteins in iron metabolism. *Plant Cell* 24 2139–2154 10.1105/tpc.112.097634 22562611 PMC3442592

[B35] Nouet C. Motte P. Hanikenne M. (2011). Chloroplastic and mitochondrial metal homeostasis. *Trends Plant Sci.* 16 395–404 10.1016/j.tplants.2011.03.005 21489854

[B36] Quinn J. M. Merchant S. (1995). Two copper-responsive elements associated with the Chlamydomonas Cyc6 gene function as targets for transcriptional activators. *Plant Cell* 7 623–628 10.1105/tpc.7.5.623 7780310 PMC160809

[B37] Raguzzi F. Lesuisse E. Crichton R. R. (1988). Iron storage in *Saccharomyces cerevisiae*. *FEBS Lett.* 231 253–258 10.1016/0014-5793(88)80742-7 3282922

[B38] Rees E. M. Thiele D. J. (2007). Identification of a vacuole-associated metalloreductase and its role in Ctr2-mediated intracellular copper mobilization. *J. Biol. Chem.* 282 21629–21638 10.1074/jbc.M703397200 17553781

[B39] Rellan-Alvarez R. Giner-Martinez-Sierra J. Orduna J. Orera I. Rodriguez-Castrillon J. A. Garcia-Alonso J. I. (2010). Identification of a tri-iron(III), tri-citrate complex in the xylem sap of iron-deficient tomato resupplied with iron: new insights into plant iron long-distance transport. *Plant Cell Physiol.* 51 91–102 10.1093/pcp/pcp170 19942594

[B40] Robinson N. J. Procter C. M. Connolly E. L. Guerinot M. L. (1999). A ferric-chelate reductase for iron uptake from soils. *Nature* 397 694–697 10.1038/17800 10067892

[B41] Santi S. Schmidt W. (2009). Dissecting iron deficiency-induced proton extrusion in *Arabidopsis* roots. *New Phytol.* 183 1072–1084 10.1111/j.1469-8137.2009.02908.x 19549134

[B42] Schagerlof U. Wilson G. Hebert H. Al-Karadaghi S. Hagerhall C. (2006). Transmembrane topology of FRO2, a ferric chelate reductase from *Arabidopsis thaliana*. *Plant Mol. Biol.* 62 215–221 10.1007/s11103-006-9015-0 16845482

[B43] Shikanai T. Muller-Moule P. Munekage Y. Niyogi K. K. Pilon M. (2003). PAA1, a P-type ATPase of *Arabidopsis*, functions in copper transport in chloroplasts. *Plant Cell* 15 1333–1346 10.1105/tpc.011817 12782727 PMC156370

[B44] Shimoni-Shor E. Hassidim M. Yuval-Naeh N. Keren N. (2010). Disruption of Nap14, a plastid-localized non-intrinsic ABC protein in *Arabidopsis thaliana* results in the over-accumulation of transition metals and in aberrant chloroplast structures. *Plant Cell Environ.* 33 1029–1038 10.1111/j.1365-3040.2010.02124.x 20132520

[B45] Sickmann A. Reinders J. Wagner Y. Joppich C. Zahedi R. Meyer H. E. (2003). The proteome of *Saccharomyces cerevisiae mitochondria. Proc. Natl. Acad. Sci. U.S.A.* 100 13207–13212 10.1073/pnas.2135385100 PMC263752 14576278

[B46] Singh A. Kaur N. Kosman D. J. (2007). The metalloreductase Fre6p in Fe-efflux from the yeast vacuole. *J. Biol. Chem.* 282 28619–28626 10.1074/jbc.M703398200 17681937

[B47] Solti Á. Kovács K. Basa B. Vértes A. Sárvári É. Fodor F. (2012). Uptake and incorporation of iron in sugar beet chloroplasts. *Plant Physiol. Biochem.* 52 91–97 10.1016/j.plaphy.2011.11.010 22305071

[B48] Sommer F. Kropat J. Malasarn D. Grossoehme N. E. Chen X. Giedroc D. P. (2010). The CRR1 nutritional copper sensor in Chlamydomonas contains two distinct metal-responsive domains. *Plant Cell* 22 4098–4113 10.1105/tpc.110.080069 21131558 PMC3027176

[B49] Tarantino D. Morandini P. Ramirez L. Soave C. Murgia I. (2011). Identification of an *Arabidopsis* mitoferrinlike carrier protein involved in Fe metabolism. *Plant Physiol. Biochem.* 49 520–529 10.1016/j.plaphy.2011.02.003 21371898

[B50] Tarantino D. Santo N. Morandini P. Casagrande F. Braun H. P. Heinemeyer J. (2010). AtFer4 ferritin is a determinant of iron homeostasis in *Arabidopsis thaliana* heterotrophic cells. *J. Plant Physiol.* 167 1598–1605 10.1016/j.jplph.2010.06.020 20724023

[B51] Terry N. Abadia J. (1986). Function of iron in chloroplasts. *J. Plant Nutr.* 9 609–646 10.1080/01904168609363470

[B52] Vasconcelos M. Eckert H. Arahana V. Graef G. Grusak M. A. Clemente T. (2006). Molecular and phenotypic characterization of transgenic soybean expressing the *Arabidopsis* ferric chelate reductase gene, FRO2. *Planta* 224 1116–1128 10.1007/s00425-006-0293-1 16741749

[B53] Vazzola V. Losa A. Soave C. Murgia I. (2007). Knockout of frataxin gene causes embryo lethality in *Arabidopsis*. *FEBS Lett.* 581 667–672 10.1016/j.febslet.2007.01.030 17258206

[B54] Vert G. Grotz N. Dedaldechamp F. Gaymard F. Guerinot M. L. Briat J. F. (2002). IRT1, an *Arabidopsis* transporter essential for iron uptake from the soil and for plant growth. *Plant Cell* 14 1223–1233 10.1105/tpc.001388 12084823 PMC150776

[B55] Vigani G. Zocchi G. Bashir K. Philippar K. Briat J. F. (2013). Signals from chloroplasts and mitochondria for iron homeostasis regulation. *Trends Plant Sci.* 18 305–311 10.1016/j.tplants.2013.01.006 23462548

[B56] Vignais P. V. (2002). The superoxide-generating NADPH oxidase: structural aspects and activation mechanism. *Cell Mol. Life Sci.* 59 1428–1459 10.1007/s00018-002-8520-9 12440767 PMC11337443

[B57] Walker E. L. Connolly E. L. (2008). Time to pump iron: iron-deficiency-signaling mechanisms of higher plants. *Curr. Opin. Plant Biol.* 11 530–535 18722804 10.1016/j.pbi.2008.06.013

[B58] Waters B. M. Blevins D. G. Eide D. J. (2002). Characterization of FRO1, a pea ferric-chelate reductase involved in root iron acquisition. *Plant Physiol.* 129 85–94 10.1104/pp.010829 12011340 PMC155873

[B59] Wintz H. Fox T. Wu Y. Y. Feng V. Chen W. Chang H. S. (2003). Expression profiles of *Arabidopsis thaliana* in mineral deficiencies reveal novel transporters involved in metal homeostasis. *J. Biol. Chem.* 278 47644–47653 10.1074/jbc.M309338200 13129917

[B60] Wu H. Li L. Du J. Yuan Y. Cheng X. Ling H. Q. (2005). Molecular and biochemical characterization of the Fe(III) chelate reductase gene family in *Arabidopsis thaliana*. *Plant Cell Physiol.* 46 1505–1514 10.1093/pcp/pci163 16006655

[B61] Yamasaki H. Hayashi M. Fukazawa M. Kobayashi Y. Shikanai T. (2009). SQUAMOSA promoter binding protein-like7 is a central regulator for copper homeostasis in *Arabidopsis*. *Plant Cell* 21 347–361 10.1105/tpc.108.060137 19122104 PMC2648088

[B62] Yamasaki K. Kigawa T. Inoue M. Tateno M. Yamasaki T. Yabuki T. (2004). A novel zinc-binding motif revealed by solution structures of DNA-binding domains of *Arabidopsis* SBP-family transcription factors. *J. Mol. Biol.* 337 49–63 10.1016/j.jmb.2004.01.015 15001351

[B63] Yi Y. Guerinot M. L. (1996). Genetic evidence that induction of root Fe(III) chelate reductase activity is necessary for iron uptake under iron deficiency. *Plant J.* 10 835–844 10.1046/j.1365-313X.1996.10050835.x 8953245

